# Synergistic redox enhancement: silver phosphate augmentation for optimizing magnesium copper phosphate in efficient energy storage devices and oxygen evolution reaction†

**DOI:** 10.1039/d3na00466j

**Published:** 2023-08-07

**Authors:** Haseebul Hassan, Muhammad Waqas Iqbal, Nora Hamad Al-Shaalan, Sarah Alharthi, Nawal D. Alqarni, Mohammed A. Amin, Amir Muhammad Afzal

**Affiliations:** a Department of Physics, Riphah International University Campus Lahore Pakistan waqas.iqbal@riphah.edu.pk; b Department of Chemistry, College of Science, Princess Nourah Bint Abdulrahman University P. O. Box 84428 Riyadh 11671 Saudi Arabia; c Department of Chemistry, College of Science, Taif University P. O. Box 11099 Taif Saudi Arabia; d Department of Chemistry, College of Science, University of Bisha Bisha 61922 Saudi Arabia

## Abstract

The implementation of battery-like electrode materials with complicated hollow structures, large surface areas, and excellent redox properties is an attractive strategy to improve the performance of hybrid supercapacitors. The efficiency of a supercapattery is determined by its energy density, rate capabilities, and electrode reliability. In this study, a magnesium copper phosphate nanocomposite (MgCuPO_4_) was synthesized using a hydrothermal technique, and silver phosphate (Ag_3_PO_4_) was decorated on its surface using a sonochemical technique. Morphological analyses demonstrated that Ag_3_PO_4_ was closely bound to the surface of amorphous MgCuPO_4_. The MgCuPO_4_ nanocomposite electrode showed a 1138 C g^−1^ capacity at 2 A g^−1^ with considerably improved capacity retention of 59% at 3.2 A g^−1^. The increased capacity retention was due to the fast movement of electrons and the presence of an excess of active sites for the diffusion of ions from the porous Ag_3_PO_4_ surface. The MgCuPO_4_–Ag_3_PO_4_//AC supercapattery showed 49.4 W h kg^−1^ energy density at 550 W kg^−1^ power density and outstanding capacity retention (92% after 5000 cycles). The experimental findings for the oxygen evolution reaction reveal that the initial increase in potential required for MgCuPO_4_–Ag_3_PO_4_ is 142 mV, indicating a clear Tafel slope of 49 mV dec^−1^.

## Abstract 2

The implementation of battery-like electrode materials with complicated hollow structures, large surface areas, and excellent redox properties is an attractive strategy to improve the performance of hybrid supercapacitors.

## Graphical abstract

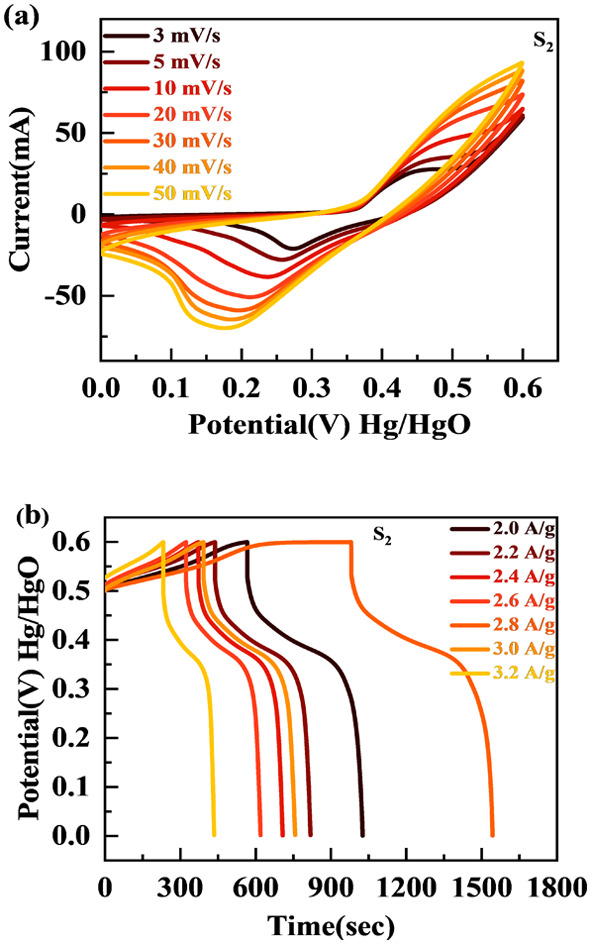

## 1. Introduction

Nowadays, the most commonly used energy resources are fossil fuels, which lead to the emission of hazardous gases into the environment. Moreover, the increasing demand for energy is resulting in the quick depletion of these energy resources. All of these circumstances require renewable energy resources to fulfill energy requirements. However, due to the intermittent behavior of wind and solar energy resources, the use of energy storage devices (ESDs) has become more prominent. An ESD balances the demands and supply of electricity. Two similar kinds of electrode are used in electric double-layer capacitors (EDLCs) that deliver quick bursts of energy, long cycle life, and exceptional power density. However, they have low energy density, limiting their ability to meet increasing technological needs.^1,2^ Lithium-ion batteries have high energy but have a disadvantage in delivering quick bursts of energy, which significantly lowers their life span compared to an EDLC. Furthermore, if overloaded or overheated, an LIB can explode.^3,4^ There have been numerous efforts to broaden the EDLC working potential and increase its energy while maintaining its power density. A new asymmetric device known as a supercapattery has recently received a lot of attention because it combines capacitive-graded materials (electrostatic storage) and other battery-graded materials (faradaic storage). RuO_2_, a well-known outstanding pseudocapacitor electrode, demonstrated an exceptional capacitance of 1340 F g^−1^. But its high expense and ecotoxicity prevent it from being used commercially. Other oxide-based metals, such as NiO and Co_3_O_4_, show promise, but their electrical resistance is low. As a result, it is critical to identify materials for a supercapattery that are inexpensive but have similar effectiveness to current materials.^5,6^ The catalysts used in the oxygen electrode for the oxygen evolution reaction (OER) have garnered significant attention due to their crucial role in these systems. There is an urgent need to develop highly efficient catalysts, free of noble metals, that exhibit superior catalytic activity for the OER. This need arises from the desire to replace expensive and scarce RuO_2_ and IrO_2_ catalysts.^7^

Scientists are presently focusing on tertiary phosphides as encouraging electrodes in supercapacitors. For example, Lei *et al.*^8^ used a simple hydrothermal process to make a ZnNiCoP–NF nanostructure, which showed 1111 C g^−1^ capacity at 10 A g^−1^. They demonstrated that the fabricated electrochemical design is ideal for energy-storage uses. Yang *et al.*^9^ produced Ni–Co–Mo ternary phosphide formed on carbon cloth fibers, which had a specific capacitance of 433 F g^−1^ at 1 A g^−1^ current density.

Silver is undoubtedly a viable substitute for carbon-based materials. Moon *et al.*^10^ developed a translucent and flexible supercapacitor with silver nanowires coated with gold. Despite their widespread use in bendable and wearable electrical devices, silver nanowire networks have high contact resistance among the nanowires.^11–13^ Silver nanoparticles with graphene, metal oxides, carbon nanotubes, and conducting polymers were tried earlier. A matrix of Ag with carbon in a composite form shows no pseudocapacitance. However, due to the increased electrical activity, its EDLC performance improves. The Ag/PANI nanocomposite synthesized by Patil *et al.*^14^ and Tang *et al.*^15^ showed that the presence of Ag in a PANI heterostructure makes the movement of ions faster. The Ag coating on PANI produces high specific energy due to quicker electron transport and electrolyte access *via* a porous network.^16^ Copper phosphate (Cu_2_PO_4_) is thought to be a perfect alternative to conventional metallic phosphates since Cu has significantly greater conductance. Cu_2_PO_4_ exhibits further intriguing dielectric and thermal characteristics. The efficacy of Cu_2_PO_4_ in supercapacitors has received little attention. Its electrochemical performance was improved by incorporating metal ions, which increases the conductivity. Thus, additional attempts to enhance the electrical conductivity of such electrode materials are required. The addition of another metal ion with similar properties can significantly improve the electrical characteristics. Thus, the movement of ions between the two metal phosphates becomes easier and the interfacial impedance of the composite substance can be reduced. Additionally, a large useful surface area is accessible for a redox reaction, increasing the capacity and power density of the supercapacitor.^17^

Herein, precipitation followed by calcination was used to produce silver phosphate nanoparticles (Ag_3_PO_4_ NPs) on amorphous MgCuPO_4_. Because of its high electrical conductivity, Ag was selected to improve the electrical conductivity of amorphous MgCuPO_4_. To prevent the recombination of particles, an amorphous MgCuPO_4_ and silver complex (Ag(NH_3_)_2_^+^) mixture was made using a sonochemical technique. MgCuPO_4_–Ag_3_PO_4_ composites were developed to raise the energy density, reduce resistance, and expand the supercapattery cycle life. The presence of low-*E*_g_ metal ions in the amorphous structure of MgCuPO_4_ is still a novel way to increase the supercapacitive performance. Furthermore, the application of the oxygen evolution reaction (OER) was also examined in the context of MgCuPO_4_–Ag_3_PO_4_ composites. The impact of different MgCuPO_4_ to Ag_3_PO_4_ weight ratios on the overall storage capability was investigated. A comprehensive charge transfer procedure between MgCuPO_4_ and solid Ag_3_PO_4_ was observed.

## 2. Experimental work

### 2.1. Materials

Carbon black, magnesium nitrate hexahydrate (Mg(NO_3_)_2_·6H_2_O), hydrochloric acid (HCl), copper(ii) nitrate tri-hydrate (Cu(NO_3_)_2_·3H_2_O), NMP, and silver nitrate (AgNO_3_) were received from Friendemann Schmidt. Tri-sodium phosphate (Na_3_PO_4_) and potassium hydroxide pellets (KOH) were bought from Duksan Pure Chemicals. All of the materials used were of analytical quality and were used without purification. Deionized water (DIW) was used throughout the experiments.

### 2.2. Synthesis of MgCuPO_4_–Ag_3_PO_4_

**Fig. 1:**
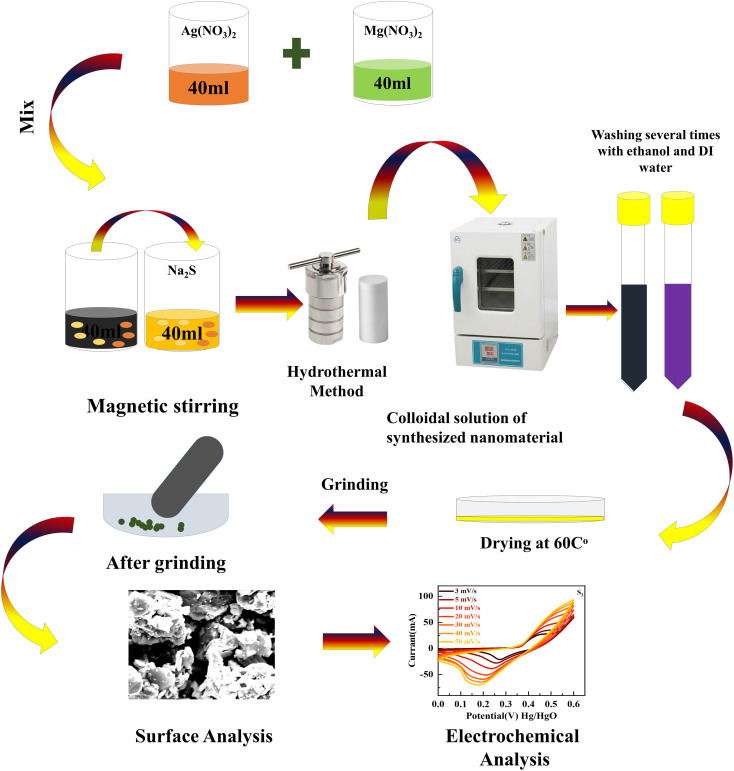
Systematic illustration of the hydrothermal technique to synthesize MgCuPO_4_–Ag_3_PO_4_ nanocomposites.

A hydrothermal and sonochemical technique was used to synthesize MgCuPO_4_–Ag_3_PO_4_ composites with various weight ratios of MgCuPO_4_ and Ag_3_PO_4_. The as-prepared MgCuPO_4_ (set A) was made using the methods described in the literature.^18^ First, 0.8 M magnesium nitrate hexahydrate (Mg(NO_3_)_2_·6H_2_O) solution was mixed with a 0.8 M solution of copper(ii) nitrate tri-hydrate (Cu(NO_3_)_2_·3H_2_O). The mixture was stirred continuously for 25 min and transferred into an autoclave and heated at 140 °C. The centrifuge machine (10 000 rpm Model RST-10M) was used to eliminate impurities from the solution. After drying at 40 °C the prepared MgCuPO_4_ was collected. The addition of NH_3_ into a 0.5 M solution of AgNO_3_ reduces Ag^+^. A horn sonicator was used for sonication until a diaminesilver(i) complex was produced. The complex solution of diaminesilver(i) was then combined with 0.02 g of MgCuPO_4_ solution and continuously stirred (3–4 h). The mixture was rinsed many times with DIW before drying for 4 h at 40 °C. Finally, the desiccated materials were calcined for 3 h at 300 °C and labeled S_1_. Two more MgCuPO_4_–Ag_3_PO_4_ nanocomposites were made for optimization reasons by altering the wt% ratio of as-prepared MgCuPO_4_ to 0.03 and 0.05 g and they were designated S_2_ and S_3_, respectively. Fig. 1 shows the synthetic process for MgCuPO_4_–Ag_3_PO_4_ nanocomposites. Table 1 shows different wt% ratios in three different composites of MgCuPO_4_–Ag_3_PO_4_ (S_1_, S_2_, and S_3_).

**Table 1:** Different weight ratios of Ag to MgCuPO_4_ for the synthesis of MgCuPO_4_–Ag_3_PO_4_ nanocomposites

Sample	MgCuPO_4_ mass	Ag mass	Weight ratio (%)
S_1_	20 mg	0.4 mg	0.083 : 1
S_2_	30 mg	0.4 mg	0.145 : 1
S_3_	50 mg	0.4 mg	0.232 : 1

### 2.3. Characterization

#### 2.3.1. Structural and surface analysis

The crystalline phases of the materials (MgCuPO_4_, S_1_, S_2_, and S_3_) were analyzed using XRD (D5000, Siemens). Kα radiation with 1.54 Å wavelength and 0.02 s^−1^ scan rate was used to determine the crystallinity of S_1_, S_2_, and S_3_. FTIR (Thermo Scientific Nicolet IS10 Smart ITR) was used to investigate the purity of the samples (S_1_, S_2_, and S_3_) which were scanned at 1 cm^−1^ resolution. X-ray photoelectron spectroscopy (XPS) measurements were taken at binding energies from 0 to 1000 eV, with 1 eV resolution. Single XPS spectra were performed at a resolution of 0.1 eV. Origin Pro 8.1 was used to fit the spectra with numerous Gaussian curves. Scanning electron microscopy (SEM) was applied to analyze the morphological characteristics of the MgCuPO_4_–Ag_3_PO_4_ nanocomposites. Jenway's 6800 design was used to determine the optical absorbance characteristics over a spectral range of 200 to 800 nm.

#### 2.3.2. Electrochemical studies

The active materials (S_1_, S_2_, and S_3_) were coated onto nickel foam (NF) and employed as a working electrode in an electrochemical research setup. In a three-cell setup, the counter and reference electrodes were platinum wire and Hg/HgO, respectively. Throughout the electrochemical studies in a typical cell, a 1 M potassium hydroxide (KOH) solution was kept constant. The slurry for the working electrode was prepared by blending 75% active material (MgCuPO_4_–Ag_3_PO_4_) and 15% carbon black, which were bonded together with the help of PVDF binder (10%). This slurry was mixed continuously for 5–6 h to produce a homogeneous suspension. This suspension was then easily applied to NF. Before starting the experiment, the NF was thoroughly cleaned with HCl, ethanol, acetone, and DIW. The NMP slurry was coated on a 1 × 1 cm^2^ area of NF. The total weight of the active material was roughly 5.5 mg. Furthermore, in the supercapattery application, activated carbon (AC) and the best composite were used as the positive and negative electrodes. The electrochemical properties of all of the materials were studied using a workstation CS300 potentiostat. A multi-channel Autolab, PGSTAT30 potentiostat was used for the Mott–Schottky (MS) measurements.

## 3. Results and discussion

### 3.1. Characterization of materials

#### 3.1.1. Mechanism of MgCuPO_4_–Ag_3_PO_4_ composite formation

The equations below were used to describe the formation mechanism of the MgCuPO_4_–Ag_3_PO_4_ nanocomposite. In the first part, a homogeneous suspension of MgCuPO_4_ nanocomposite was formed using a hydrothermal approach. After that positive Ag(NH_3_)_2_^+^ was electrostatically bound with the phosphate group and arranged around MgCuPO_4_. The presence of an excess amount of H_3_PO_4_^2−^ in the as-synthesized MgCuPO_4_ causes the quick development of a homogeneous MgCuPO_4_–Ag_3_PO_4_ nanocomposite after stirring. Ammonium (NH_4_^+^) and other unwanted impurities were eliminated upon calcination.

MgCuPO_4_ formation1
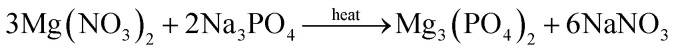
2
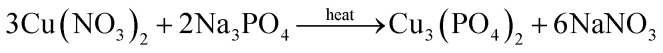
3
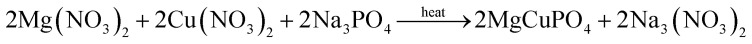


MgCuPO_4_–Ag_3_PO_4_ formation4
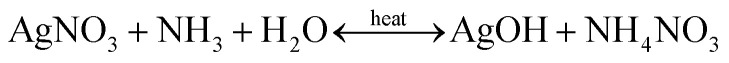
5
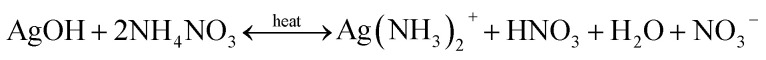
6
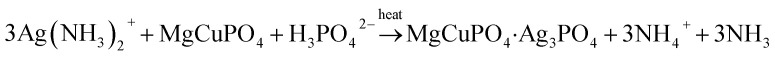


#### 3.1.2. Structural analysis of MgCuPO_4_–AgPO_4_

**Fig. 2:**
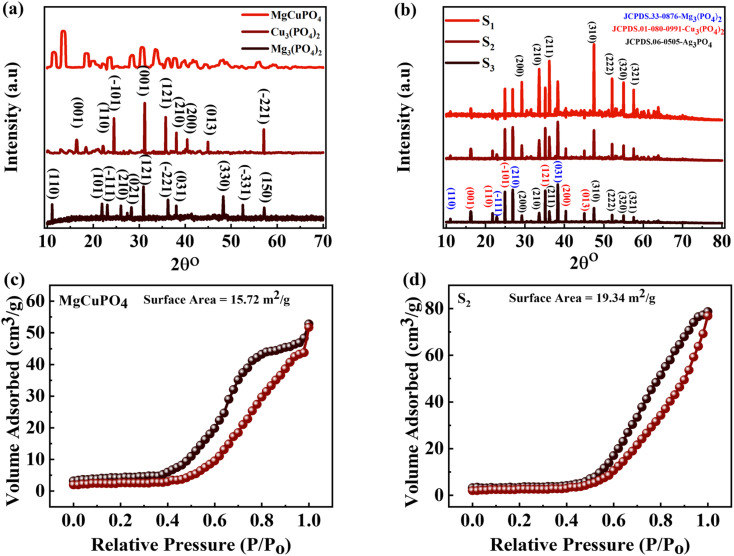
(a) The XRD pattern for Mg_3_(PO_4_), Cu_3_(PO_4_), and MgCuPO_4_. (b) The XRD pattern for MgCuPO_4_–Ag_3_PO_4_ (S_1_, S_2,_ S_3_) nanocomposites. (c and d) BET measurement for MgCuPO_4_ and the S_2_ nanocomposite.

The crystallinities of MgCuPO_4_, S_1_, S_2_, and S_3_ were studied by XRD and the outputs are depicted in Fig. 2. First, the XRD of MgCuPO_4_ was studied, as indicated by Fig. 2(a). There were three different XRD patterns represented by three different colors assigned to Mg_3_(PO_4_)_2_, Cu_3_(PO_4_)_2_, and MgCuPO_4_. The diffraction spikes at 2*θ* = 12°, 22°, 23.2°, 26°, 28.32°, 31.3°, 36.5°, 38°, 48.23°, 52.8°, and 57.1° belong to the (110), (101), (−111), (210), (021), (121), (−221), (031), (330), (−331), and (150) planes, which match JCPDS 33-0876.^19^ The XRD peaks for Cu_3_(PO_4_)_2_ showed diffraction spikes at 2*θ* = 15.32°, 21.2°, 25°, 31.33°, 35°, 37°, 40°, 44.3°, and 56.5° belonging to (001), (110), (−101), (001), (121), (210), (200), (013), and (−221), which matches JCPDS 01-080-0991.^20^ The XRD pattern for MgCuPO_4_ indicated almost similar diffraction patterns to Mg_3_(PO_4_)_2_ and Cu_3_(PO_4_)_2_. The width of these spikes was evidence of the amorphous nature of MgCuPO_4_. The incorporation of Ag_3_PO_4_ is indicated in Fig. 2(b). Three different XRD patterns for S_1_, S_2_, and S_3_ are represented in Fig. 2(b). There were multiple reflections associated with S_1_, S_2_, and S_3_. The XRD pattern shows the crystalline nature of Ag_3_PO_4_. The strong peaks for Ag_3_PO_4_ at 2*θ* = 29.1°, 33.3°, 36.6°, 47.5°, 52.1°, 55.0°, and 57.5° correspond to the (200), (210), (211), (310), (222), (320), and (321) planes. This XRD pattern correlated with JCPDS 06-0505, which shows the formation of Ag_3_PO_4_.^21^ As the amount of MgCuPO_4_ decreased, the intensity of XRD spikes for Ag_3_PO_4_ increased. The lack of a positional shift in the Ag_3_PO_4_ peaks indicates that Ag_3_PO_4_ was not incorporated into the MgCuPO_4_ lattice, but rather developed on the MgCuPO_4_ surface.^22^Table 2 shows the lattice parameters of the samples.

**Table 2:** Structural parameters for the analysis of MgCuPO_4_ and MgCuPO_4_–Ag_3_PO_4_ nanocomposites

Sample	Lattice parameters			Volume (nm)^3^	Particle size (nm)	Strain (*ε*_r_) (10^−3^)	Density (g cm^−3^)	Dislocation density (10^15^ m^−2^)
	*α*	*β*	*Γ*					
Standard	7.599	8.235	5.076					
MgCuPO_4_	7.597	8.237	5.075	0.38	40–50	0.65	5.4087	0.55
S_1_	7.600	8.240	5.075	0.41	51–56	0.71	5.4093	0.58
S_2_	7.613	8.243	5.074	0.43	53–58	0.69	5.4098	0.63
S_3_	7.615	8.247	5.074	0.44	52–59	0.70	5.4100	0.71

The specific surface areas (SSAs) of MgCuPO_4_ and the MgCuPO_4_–Ag_3_PO_4_ (S_2_) nanocomposite were calculated using the Brunauer–Emmett–Teller (BET) technique, because S_2_ demonstrated the best performance compared to S_1_ and S_3_, as described in the following part. The N_2_ adsorption–desorption isotherms of MgCuPO_4_ and S_2_ are shown in Fig. 2(c) and (d). The BET graphs for MgCuPO_4_ and S_2_ exhibit an IV-type hysteresis loop, showing their porous structure. The porous behavior of MgCuPO_4_ and S_2_ resulted in decreases in electrolyte ion diffusion time. The SSA of MgCuPO_4_ and S_2_ were 15.72 and 19.34 m^2^ g^−1^, respectively. The total pore volumes for MgCuPO_4_ and S_2_ estimated through BET calculations were 0.04928 and 0.052596 cm^3^ g^−1^, respectively. The smaller SSA and pore volume of MgCuPO_4_ compared to S_2_ were due to the absence of Ag_3_PO_4_ in the MgCuPO_4_–Ag_3_PO_4_ nanocomposite. Furthermore, this may be due to the distribution of Ag_3_PO_4_ NPs into MgCuPO_4_.^23^

**Fig. 3:**
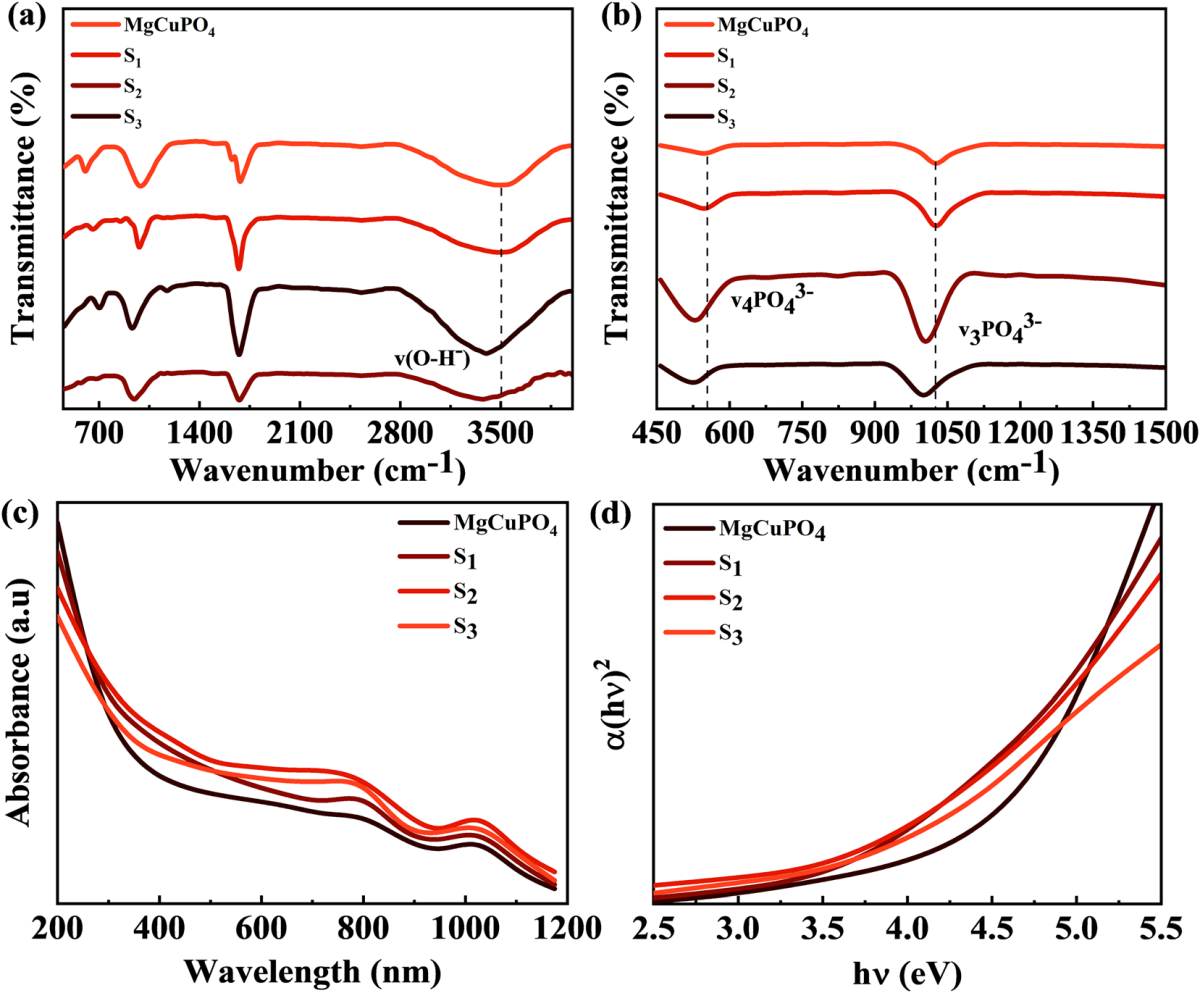
(a) FTIR spectra for MgCuPO_4_, S_1_, S_2_, and S_3_ at 500–4000 cm^−1^. (b) FTIR spectra for MgCuPO_4_, S_1_, S_2_, and S_3_ at 450–1500 cm^−1^. (c) UV-vis spectra for MgCuPO_4_, S_1_, S_2_, and S_3_. (d) The band gap determination for MgCuPO_4_, S_1_, S_2_, and S_3_.

Fig. 3(a) and (b) show the FTIR spectra of amorphous MgCuPO_4_, S_1_, S_2_, and S_3_. The band for the O–P–O bond (*v*_4_ PO_4_^3−^) for the triply degenerate asymmetric mode occurred at 563 cm^−1^. While the band at 1036 cm^−1^ belongs to the P–O bond (*v*_3_ PO_4_^3−^) stretching mode. The above-mentioned bands are evidence of the presence of phosphate (PO_4_^3−^). The bands moved to a lower wavenumber as the amount of Ag_3_PO_4_ increased in the MgCuPO_4_–Ag_3_PO_4_ nanocomposites.^24^ The band appearing at 1645 cm^−1^ belongs to *v*_1_ (A_1_) H_2_O and those at 3200–3800 cm^−1^ belong to *v*_2_ (A_1_) H_2_O. After the incorporation of Ag_3_PO_4_ (S_1,_ S_2_, and S_3_), the band intensities rose considerably, showing that the percentages of phosphorous and water on the MgCuPO_4_–Ag_3_PO_4_ surface increased.^25^ The adsorbed water resulted in increased storage performance due to an enhanced inter-particle path.^26,27^

#### 3.1.3. Optical properties of MgCuPO_4_–AgPO_4_

UV-vis absorption spectroscopy was applied to check the impact of Ag_3_PO_4_ on the optical performance of MgCuPO_4_. The spectra are shown in Fig. 3(c). In a deformed octahedral coordinated environment, MgCuPO_4_ has an absorption edge at 808 nm and a second absorption edge at 1000 nm, which are due to Cu^+^ and Mg^+^, respectively.^28,29^ Because of the decreasing fractions of MgCuPO_4_ found in MgCuPO_4_–Ag_3_PO_4_ nanocomposites, the absorptivity of MgCuPO_4_ progressively declines from S_1_ to S_3_. Furthermore, as the proportions of MgCuPO_4_ decreased, the absorption edge red shifted towards the visible region. The existence of more Ag_3_PO_4_ NPs than MgCuPO_4_, which absorbs in the visible region, causes the peaks to redshift. This redshift may also be due to narrowing of the band gap, as evidenced by the altered Kubelka–Munk function of light energy (*αhν*)^2^*versus* photon energy (*hν*), as shown in Fig. 3(d). The Tauc equation was used to determine the band gap (*E*_g_) values of the samples.^30,31^

7 (*αhν*)^2^ = *K*(*hν* − *E*_g_)

The absorption coefficient is denoted by *α* and the proportionality constant by *K*. *K* was determined from *K* = (2.303 × 10^3^)(*A*)/*L*, where absorbance is represented by *A* and optical path by *L* (1 cm). The *E*_g_ value was estimated by extrapolating the straight line of the image shown in Fig. 3(d). *E*_g_ values for S_1_, S_2_, and S_3_ and amorphous MgCuPO_4_ were 3.9, 3.6, 3.5, and 4.3 eV, respectively. The electrical insulating properties of amorphous MgCuPO_4_ were reflected in its high *E*_g_ value. The gradual drop in *E*_g_ in MgCuPO_4_–AgPO_4_ nanocomposites was due to the combined impact between the two elements. The overlapping of energy levels causes a reduction in the band gap according to energy band theory. This indicates that Ag_3_PO_4_ were well developed on the MgCuPO_4_ surface, exhibiting the combined electronic characteristics.^32,33^

#### 3.1.4. XPS spectrum for MgCuPO_4_–AgPO_4_

**Fig. 4:**
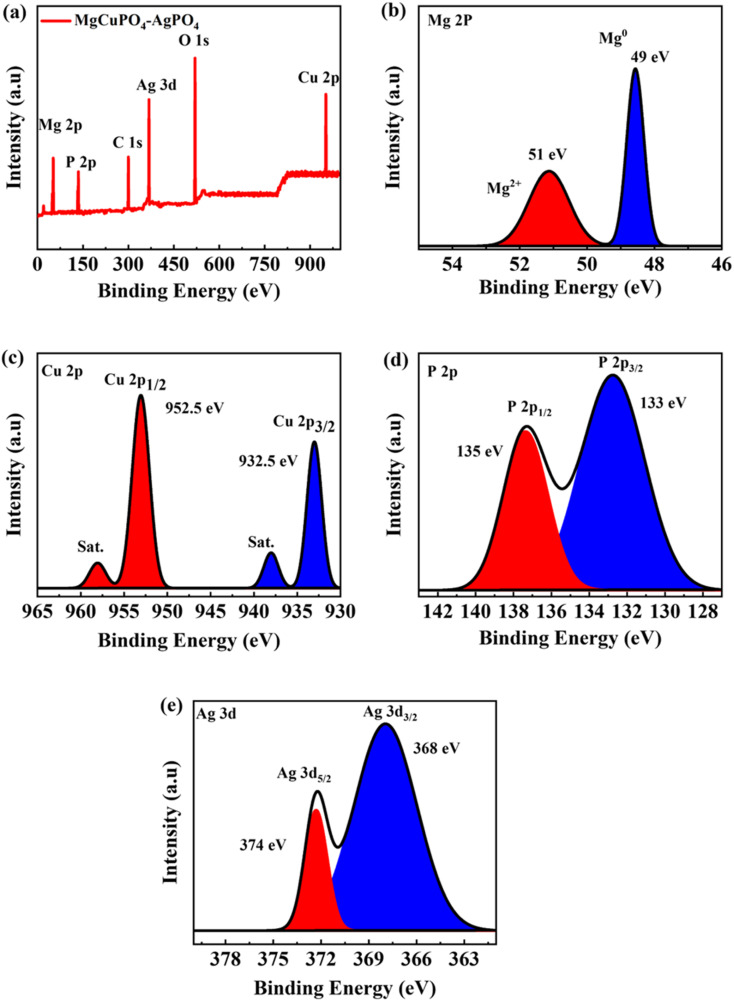
(a) XPS survey spectrum for S_2_. (b) XPS spectrum for Mg. (c) XPS spectrum for Cu 2p. (d) XPS spectrum for P 2p. (e) XPS spectrum for Ag 3d.

Because of the dispersion of Ag_3_PO_4_ NPs on the MgCuPO_4_ surface, XPS was used to analyze the surface composition of MgCuPO_4_ and MgCuPO_4_–AgPO_4_ nanocomposites. The XPS spectrum of S_2_ showed the presence of several distinct peaks due to Mg, P, C 1s, Ag, O 1s, and Cu, as indicated in Fig. 4(a). Fig. 4(b) depicts the deconvoluted core level spectrum of Mg 2p, which includes two bands at 49 and 51 eV binding energy corresponding to Mg^2+^ and Mg^0^, respectively.^34^ The XPS spike for Cu 2p_3/2_ located at 932.5 eV correlates to Cu^2+^ possibly reacting with a phosphate ion. The peak for Cu 2p_1/2_ appeared at 952.5 eV, as indicated in Fig. 4(c).^35^ The XPS spectrum for P 2p is represented in Fig. 4(d). The presence of P 2p_1/2_ at 135 eV and P 2p_3/2_ at 133 eV confirm the presence of phosphorous. The deconvoluted XPS spectrum of P 2p showed that the first peak at 133 eV accounts for 75–80% of the P 2p signal. This can be ascribed to the oxidation of the MgCuPO_4_ surface metal phosphate molecule in PO_4_^3−^.^36^ The second spike at 135 eV showed the presence of 10–20% residual metaphosphate.^37^ S_2_ demonstrated the presence of symmetrical spin–orbit components. The XPS spectra for Ag showed two distinct peaks which were separated by 6 eV. The Ag 3d_5/2_ peak appeared at 368 eV and Ag 3d_3/2_ peak appeared at 374 eV, which corresponds to Ag^+^, as indicated in Fig. 4(e).^38^

#### 3.1.5. Morphological study of MgCuPO_4_–AgPO_4_

**Fig. 5:**
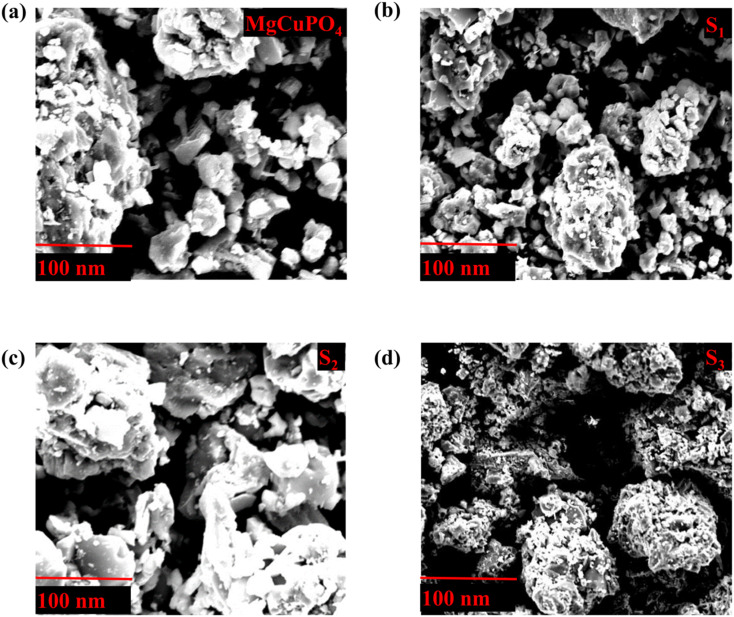
(a–d) SEM images for S_1_, S_2_, S_3_, and S_4_ composites.

SEM analysis was used to examine the shape of MgCuPO_4_ and its three composites with Ag_3_PO_4_ (S_1,_ S_2_, and S_3_). Fig. 5(a)–(d) depict an uneven form of amorphous MgCuPO_4_ and its composites with Ag_3_PO_4_ with 40–50 nm particle size. Fig. 5 shows that as the amorphous MgCuPO_4_ content of the nanocomposite decreased, more Ag_3_PO_4_ NPs were found on the surface of the amorphous MgCuPO_4_. However, for S_1_ and S_3_, the recombination of ions could restrict the diffusion of ions. The recombination of ions could be attributed to an overabundance of Ag_3_PO_4_, which restricts the movement of MgCuPO_4_ ions.^39,40^

### 3.2. Electrochemical results

**Fig. 6:**
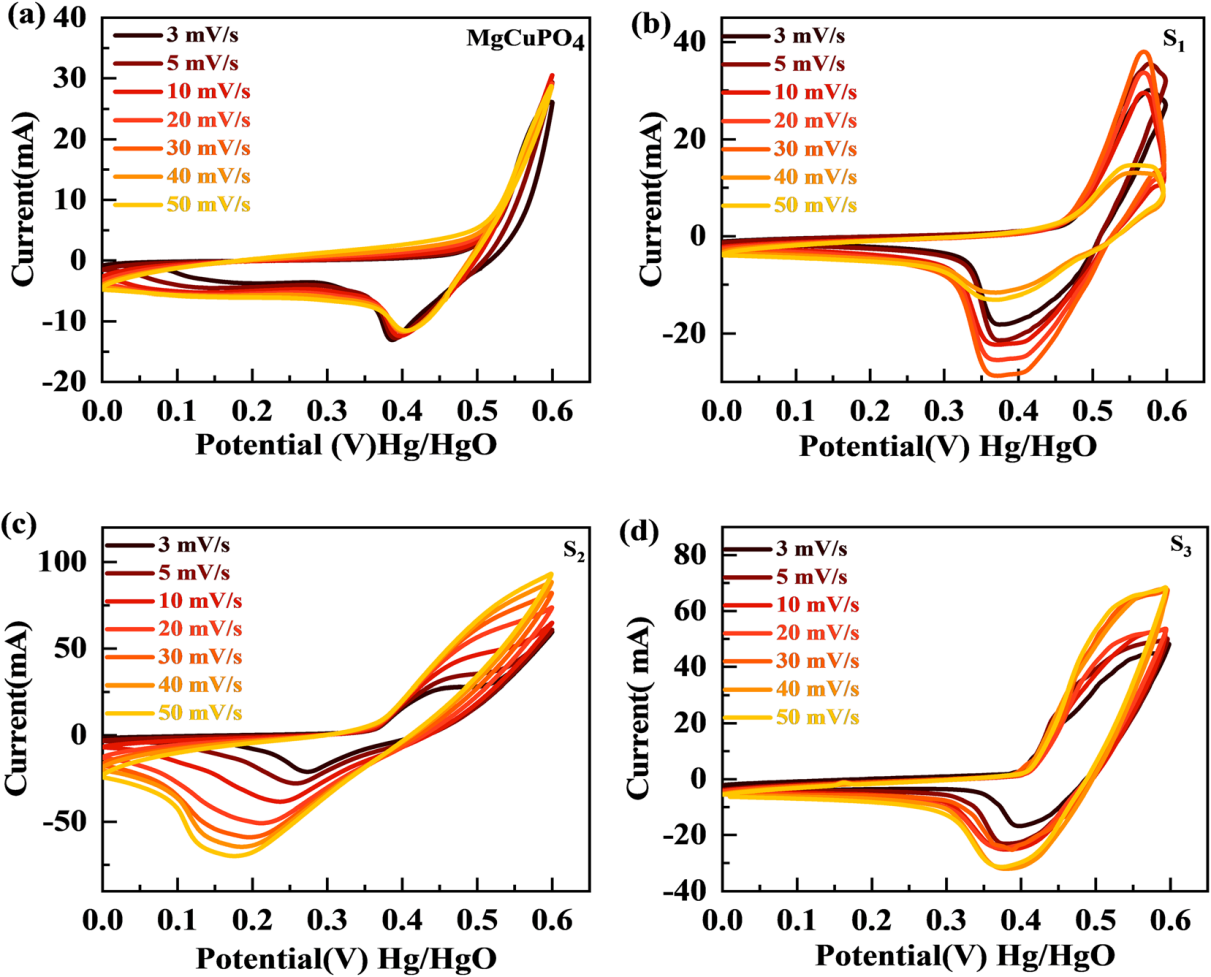
(a–d) The CV curves for MgCuPO_4_, S_1_, S_2_, and S_3_, respectively.

The synthesized samples (MgCuPO_4_, S_1_, S_2_, and S_3_) were then electrochemically tested using CV measurements, which were determined in the potential window (PW) of 0–0.6 V at a scan rate of 3–50 mV s^−1^, as depicted in Fig. 6. For all samples, faradaic behavior was observed from the presence of redox peaks. This was because of the faster movement of ions within the materials, causing a redox reaction at the surface of the electrodes.^41,42^ S_2_ had the greatest redox current strength at 3 mV s^−1^ scan rate compared to the other MgCuPO_4_–AgPO_4_ nanocomposites. This shows that the addition of Ag_3_PO_4_ increased the redox current due to the synergetic impact of MgCuPO_4_ and Ag_3_PO_4_. But further addition of Ag_3_PO_4_ influenced the redox potentials, as can be seen from the CV plot of S_3_ (Fig. 6(d)). The finding suggested that electrolyte ions engage extensively with the numerous electro-active sites of the materials when the amount of Ag_3_PO_4_ increases. With higher loadings of Ag_3_PO_4_ in MgCuPO_4_, the electrical transmission route and electrolyte ion diffusion rate of the nanocomposites differ. This indicated that the number of phosphate groups in the MgCuPO_4_–Ag_3_PO_4_ composite, as well as metal ion concentrations, affect electron and ion motion.^43^ Furthermore, the variations in anodic and cathodic potentials can be linked to the presence of the redox process.^44^ The CV curve of MgCuPO_4_ changes its shape at 100 mV s^−1^ (Fig. 6(a)) because the electrolyte ions did not reach the MgCuPO_4_ inner sites.^45^ S_2_ had a greater CV area (and thus a greater specific capacity) than MgCuPO_4_ at a low scan rate. S_2_ also maintained its form at higher scan rates due to its stable behavior.^46,47^Fig. 6(a)–(d) illustrate the CV curves for S_2_ and the other samples (MgCuPO_4_, S_1_, and S_3_) that were gradually enhanced with rising scan rates (3–50 mV s^−1^). Fig. S1† shows the CV curves for MgCuPO_4_, S_1_, and S_3_ at an operating potential of 0–0.8 V.

**Fig. 7:**
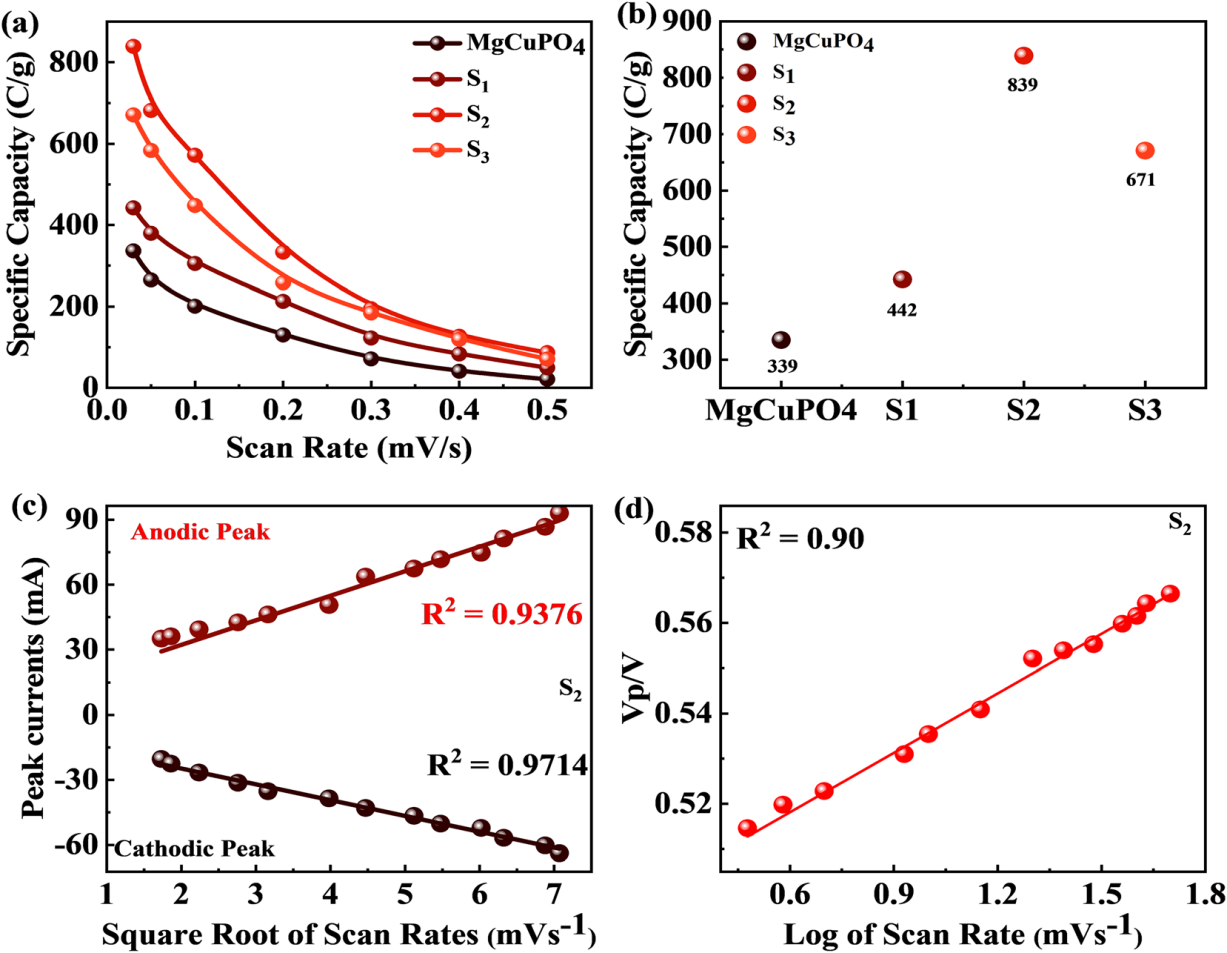
(a) Specific capacity for MgCuPO_4_, S_1_, S_2_, and S_3_ at various scan rates. (b) Specific capacity for MgCuPO_4_, S_1_, S_2_, and S_3_ at 3 mV s^−1^ scan rate. (c) Anodic and cathodic peaks calculated for S_2_. (d) Peak voltage plotted against log of scan rate for S_2_.

For battery-type materials, the most suitable word to determine the storage capability is the specific capacity (C g^−1^). However, some researchers in the literature also use capacitance (F g^−1^) which is not correct. Table 3 shows the values of specific capacity at all scan rates. Eqn (8) can be used to determine the specific capacity.^48^8
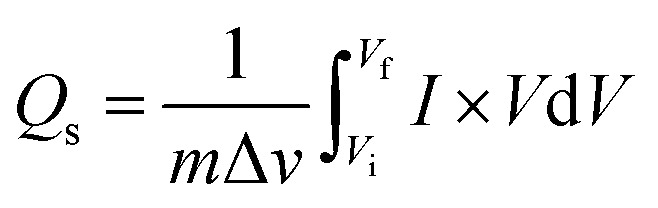
In eqn (8), *Q*_s_ indicates the specific capacity, *m* is the active mass, Δ*v* represents the change in scan rate; the current is denoted by *I*, and the operating potential by *V*. The specific capacities calculated for MgCuPO_4_, S_1_, S_2_, and S_3_ at various scans are represented in Fig. 7(a). The specific capacities for MgCuPO_4_–Ag_3_PO_4_ nanocomposites were higher than that for MgCuPO_4_. The specific capacity for S_2_ at 3 mV s^−1^ was 839 C g^−1^, which was higher than that for MgCuPO_4_ (335 C g^−1^), S_1_ (442 C g^−1^), or S_3_ (671 C g^−1^). The specific capacities for all samples (MgCuPO_4_, S_1_, S_2_, and S_3_) at 3 mV s^−1^ are represented in Fig. 7(b). When an amount of Ag_3_PO_4_ is added into the heterostructure of MgCuPO_4_, the specific capacity increases due to the combined impact of MgCuPO_4_–Ag_3_PO_4_, with a large number of accessible pores and faster movement of ions. A further increase in the amount of Ag_3_PO_4_ resulted in a decrease in its capacity. An excess amount of Ag_3_PO_4_ also results in blockage of a number of pores. Therefore, the specific capacity for S_3_ decreases.

**Table 3:** Specific capacity for MgCuPO_4_, S_1_, S_2_, and S_3_ through CV and GCD

Scan rate (mV s^−1^)	Specific capacity (C g^−1^)							
	MgCuPO_4_		S_1_		S_2_		S_3_	
	CV	GCD	CV	GCD	CV	GCD	CV	GCD
3	336.16	317	442	330	839	1138	671	512
5	264.60	269	379.71	269	682.04	933	583.42	478
10	200.12	245	305.08	251	571.73	891	447.96	447
20	129.15	215	212.86	236	333.16	832	257.97	426
30	71.05	192	122.49	225	194.76	745	184.12	398
40	40.49	183	83.07	210	126.813	682	119.18	355
50	20.88285	169	49.40	192	86.44	663	70.54	313

The positive and negative current spikes measured through CV readings of S_2_ gradually increased with the square root of the scan rate. The value of the *I*_pa_/*I*_pc_ fraction was almost 1. This indicated that the reversible faradaic processes were responsible for charge storage, as shown in Fig. 7(c).^49^ The linear relation between anodic and cathodic currents was evidence of the battery nature of the MgCuPO_4_–Ag_3_PO_4_ (S_2_) nanocomposite. Fig. 7(d) indicates a straight-line relationship between voltage and log of scan rate, which proves the diffusion-controlled process for S_2_. The chemical equations below can be used to describe redox reactions.^50^9
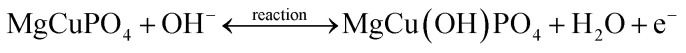
10
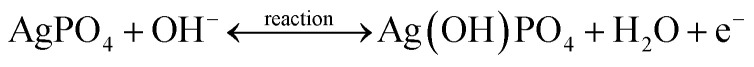


**Fig. 8:**
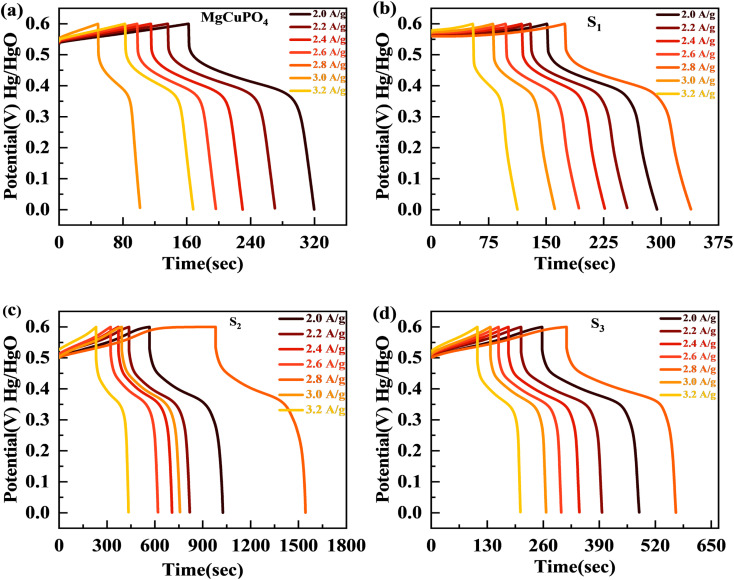
(a–d) The GCD plots for MgCuPO_4_, S_1_, S_2_, and S_3_ at different currents.

**Fig. 9:**
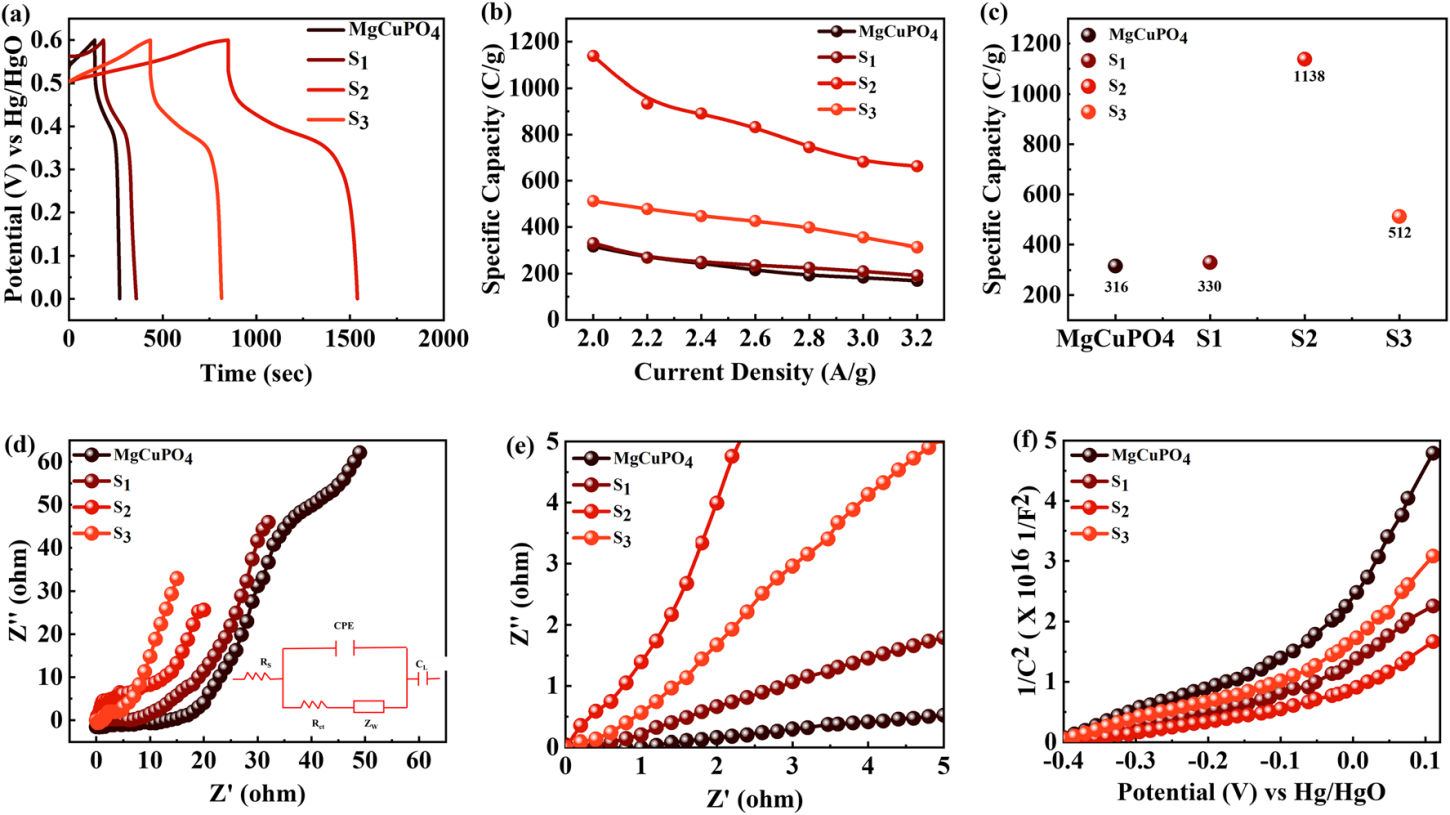
(a) GCD comparison for MgCuPO_4_, S_1_, S_2_, and S_3_ at 2.0 A g^−1^ current density. (b) Specific capacity computed for MgCuPO_4_, S_1_, S_2_, and S_3_ at varied current densities. (c) Specific capacity computed for MgCuPO_4_, S_1_, S_2_, and S_3_ at 2 A g^−1^ current density. (d) EIS graph for MgCuPO_4_, S_1_, S_2_, and S_3_. (e) High-magnification EIS images for MgCuPO_4_, S_1_, S_2_, and S_3_. (f) MS plot for MgCuPO_4_, S_1_, S_2_, and S_3_.

The discharge profiles of MgCuPO_4_ and MgCuPO_4_–Ag_3_PO_4_ nanocomposites obtained using the GCD method are shown in Fig. 8(a)–(d). The shape of the GCD curves was non-linear for MgCuPO_4_, S_1_, S_2_, and S_3_ because of deep ion interaction, showing that charge storage contribution comes mainly from the redox reactions. At a current density of 2 A g^−1^, S_2_ had the greatest discharge time compared to the other MgCuPO_4_–AgPO_4_ nanocomposite electrodes. At 3.2 A g^−1^ current, the GCD trajectory of MgCuPO_4_ was also relatively shorter than that of S_2_. This showed that the performance of MgCuPO_4_ decreases with current density more frequently than the other MgCuPO_4_–Ag_3_PO_4_ nanocomposites, as illustrated by Fig. 8(a)–(d). The GCD comparison at 2.0 A g^−1^ for MgCuPO_4_, S_1_, S_2_, and S_3_ is represented in Fig. 9(a). As can be seen from Fig. 9(a), S_2_ had the largest plateau region compared to the other samples. This may be due to the larger surface area exposed to electrolytic ions, faster transportation of ions, and higher conductivity of S_2_. The specific capacity, *Q*_s_, of the electrodes was determined using eqn (11) from the galvanostatic discharge data.11
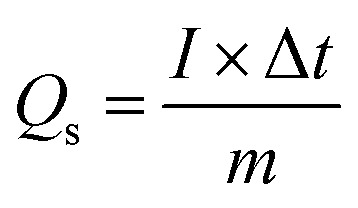
in eqn (11), *Q*_s_ represents the specific capacity, *I* is the current, Δ*t* indicates the discharge time, and *m* is the active mass. Fig. 9(b) depicts a plot of specific capacity for MgCuPO_4_, S_1_, S_2_, and S_3_ against current density derived from the GCD curves. At all present current densities, the specific capacity for MgCuPO_4_ had reduced from 316 (2 A g^−1^) to 169 C g^−1^ (3.2 A g^−1^), with 53% capacity retention. S_1_ had a capacity retention of 58% with a capacity of 330 C g^−1^ at 2 A g^−1^ and 192 C g^−1^ at 3.2 A g^−1^. Again S_2_ outperforms the other composite materials, showing 1138 C g^−1^ capacity at 2 A g^−1^. S_2_ can retain 663.8 C g^−1^ capacity at 3.2 C g^−1^ showing 59% capacity retention. Furthermore, an increase in the wt% ratio of Ag_3_PO_4_ decreases the overall performance. The specific capacities decrease for S_3_ to 512 C g^−1^ at 2 A g^−1^ and 313.6 C g^−1^ at 3.2 A g^−1^, demonstrating 61% capacity retention.

This increase in rate capacity was due to the presence of Ag_3_PO_4_ NPs, which decreased the internal impedance of MgCuPO_4_.^51^ Among all the MgCuPO_4_–Ag_3_PO_4_ (S_1_, S_2_, and S_3_) nanocomposites studied, S_2_ had the highest capacity. The increased electrochemical performance for S_2_ was due to two conflicting effects: raising the quantity of Ag_3_PO_4_ increases the conductivity of MgCuPO_4_–Ag_3_PO_4_ nanocomposites. However, too many Ag_3_PO_4_ nanoparticles degrade the performance of the S_3_ electrode due to agglomerations produced, which reduces the contact area between the electrode and electrolytic solution. Another cause was the lack of amorphous MgCuPO_4_ in S_3_, as amorphous MgCuPO_4_ was responsible for increased active sites for redox reactions.^52^ Therefore, owing to the structural stability of crystalline Ag_3_PO_4_ NPs, the rate capacity of S_2_ remains greater than that of MgCuPO_4_. Fig. 9(c) indicates the comparative calculated specific capacities for MgCuPO_4_, S_1_, S_2_, and S_3_ at 2.0 A g^−1^.

The electrochemical impedance spectra (EIS) of the electrode materials were measured to determine charge and ion transport. The Nyquist graphs of amorphous MgCuPO_4_, S_1_, S_2_, and S_3_ are shown in Fig. 9(d). Because of the roughness of the electrode surface, uneven electric field, and varying electrochemical activity, the form of the EIS plots departs from the semicircular shape.^53^ The corresponding series resistance was calculated using the contact of the EIS graphs with the real part of the impedance spectrum. At high frequencies, the semicircle width is attributed to charge transfer impedance (*R*_ct_).^54^ In Fig. 9(d) (inset), the circuit diagram for the fitted Nyquist plot is also represented. The semicircle can be seen from the ZCPE and *R*_ct_ parallel connection in the circuit diagram. According to the EIS diagram, the *R*_s_ values for S_1_, S_2_, S_3_, and MgCuPO_4_ were 0.72, 0.65, 0.81, and 1.05 U, respectively, as shown in Fig. 9(d). The lower *R*_s_ values of all MgCuPO_4_–AgPO_4_ nanocomposites compared to amorphous MgCuPO_4_ nanocomposites suggest higher conductance. Moreover, the semicircle diameter of MgCuPO_4_ was considerably bigger than that of the MgCuPO_4_–AgPO_4_ nanocomposites in the Nyquist plots, which results in greater charge transfer resistance. In all the MgCuPO_4_–Ag_3_PO_4_ nanocomposites studied, S_2_ had the shortest diameter, indicating the lowest internal resistance. Furthermore, the straight line for S_2_ was the sharpest, indicating the lowest ion diffusion resistance. The high-resolution EIS spectra for MgCuPO_4,_ S_1_, S_2,_ and S_3_ are represented in Fig. 9(e). A potential-dependent capacity test was used to further explore the effect of Ag_3_PO_4_ modification on the electrical characteristics of MgCuPO_4_. MS plots were made from the capacitances obtained from the imaginary portion of the impedance. Fig. 9(f) depicts the MS plots for MgCuPO_4_, S_1_, S_2,_ and S_3_. The dominant charge carriers in all MgCuPO_4_, S_1_, S_2_, and S_3_ materials were electrons, as indicated by the positive slope of the straight lines (n-type semiconductors). The slope obtained from the straight line plotted between 1/*C*^2^ and *V* can be used to evaluate charge carrier density among electrode materials. The charge carrier has a negative relationship with the MS inclination.^55^ The S_1_, S_2_, and S_3_ slopes were lower than those of MgCuPO_4_, implying that the MgCuPO_4_–Ag_3_PO_4_ composites had a greater charge carrier density. The S_2_ curve had the lowest slope, demonstrating its increased electron density. These findings suggest that the incorporation of Ag_3_PO_4_ into MgCuPO_4_ results in a higher density of charge carriers.^56^

The electron transmission rate of crystalline Ag_3_PO_4_ NPs differs from that of diffuse MgCuPO_4_. The number of pores provided by MgCuPO_4_ was used to store the electrolyte ions that were able to penetrate the inner surface of MgCuPO_4_. Most of the amorphous MgCuPO_4_ surfaces were engaged in the faradaic reactions. Additionally, the electrolyte could not reach internal surfaces at a higher scan rate/current density, thus producing a low specific capacity.^57^ In contrast, the electrolytic ions interact with the topmost surface layer of Ag_3_PO_4_ NPs as well as the inner surface of MgCuPO_4_. Thus, Ag_3_PO_4_ adds useful surface area to unutilized amorphous MgCuPO_4_ surfaces and maintains a large number of oxidation/reduction reactions at higher currents.^58^ Despite the fact that amorphous MgCuPO_4_ offers a porous structure that helps to store more electrolytic ions, it still had low conductance.^59^ In this study, the high electrical conductivity of Ag_3_PO_4_ NPs fixed on the weakly conductive amorphous MgCuPO_4_ accelerates the electron transfer mechanism and lowers the impedance of the MgCuPO_4_–Ag_3_PO_4_ composites. Furthermore, the Ag_3_PO_4_ nanoparticles reduce the distance between electron transport routes and the collector, resulting in increased electrochemical performance.^60^

### 3.3. Supercapattery

**Fig. 10:**
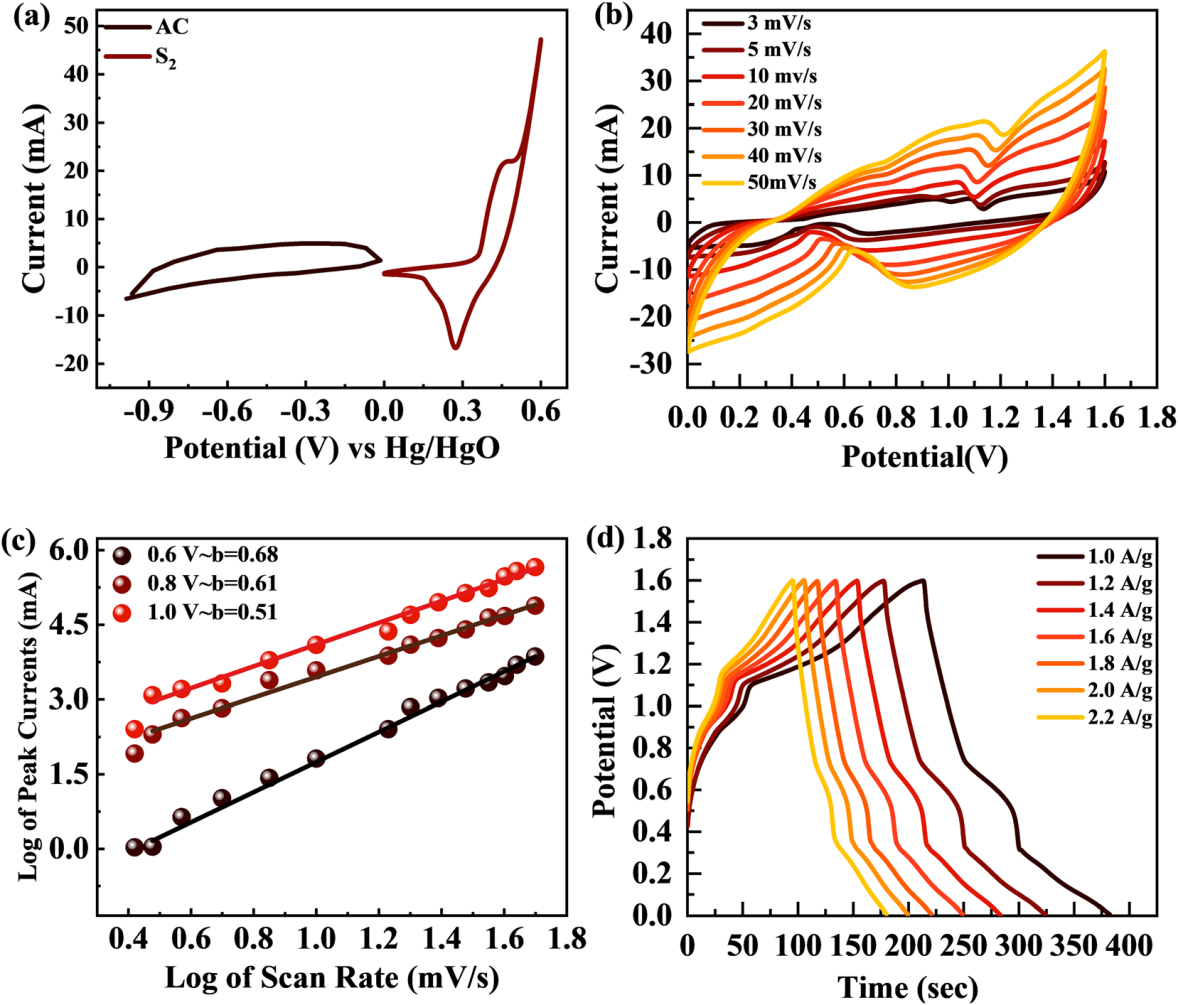
(a) CV curves for AC and S_2_ at 3 mV s^−1^. (b) CV curves for the S_2_//AC supercapattery at 3–50 mV s^−1^. (c) *b*-fitting computed for the S_2_//AC device. (d) GCD curves for the S_2_//AC supercapattery at 1–2.2 A g^−1^ current density.

The broad PW results in increased energy density for SCs. The supercapattery (S_2_//AC) was designed with S_2_ and activated carbon (AC). AC due to its permeable and porous structure allows more electrolyte ions to be stored.^61^ The individual CV graphs were originally measured in a three-cell setup for S_2_ and AC to measure the overall voltage of the supercapattery (Fig. 10(a)). The working PW of AC was 0 to −1 V and that of S_2_ was 0–0.6 V. Thus, the S_2_//AC supercapattery was stable over 1.6 V. No redox peak appeared at 0–0.5 V, which showed that the dominant storage mechanism was provided by the capacitive nature of AC. At higher potential, >0.6 V, redox peaks emerged, indicating the faradaic reaction of S_2_. Fig. 10(b) shows that the shape of the CV graphs for the S_2_//AC supercapattery was not altered at a higher scan (100 mV s^−1^), which proves the high stability of the S_2_//AC device.

The power law, which connects current density *I* with scan rate (*v*), was used for theoretical study of the electrochemical behavior of the asymmetric device. The charge storage process in a supercapattery is investigated using *b*-fitting. The *b*-value was calculated using the slope drawn between the log of scan rate and the log of peak current.

12 *I* = *kv*^*b*^

13 ln *I* = ln *k* + *b* ln *v*

The adjustable factors are represented by *k* and *b*, as stated in the equations, while *v* is the scan rate and *I* is the current. The exponent *b* is critical in deciding the charge accumulation in a supercapattery. For a *b*-value of 0–0.5, the dominant charge storage process is diffusion regulated. For a *b*-value of 0.8–1.0, the dominant charge storage process is adsorption/desorption. For a *b*-value of 0.5–0.8, the storage process is due to both faradaic and adsorption/desorption processes.^62^ The *b*-fitting for this supercapattery (S_2_//AC) was between 0.5 and 0.8, as shown in Fig. 10(c). This proves the formation of a supercapattery.

**Fig. 11:**
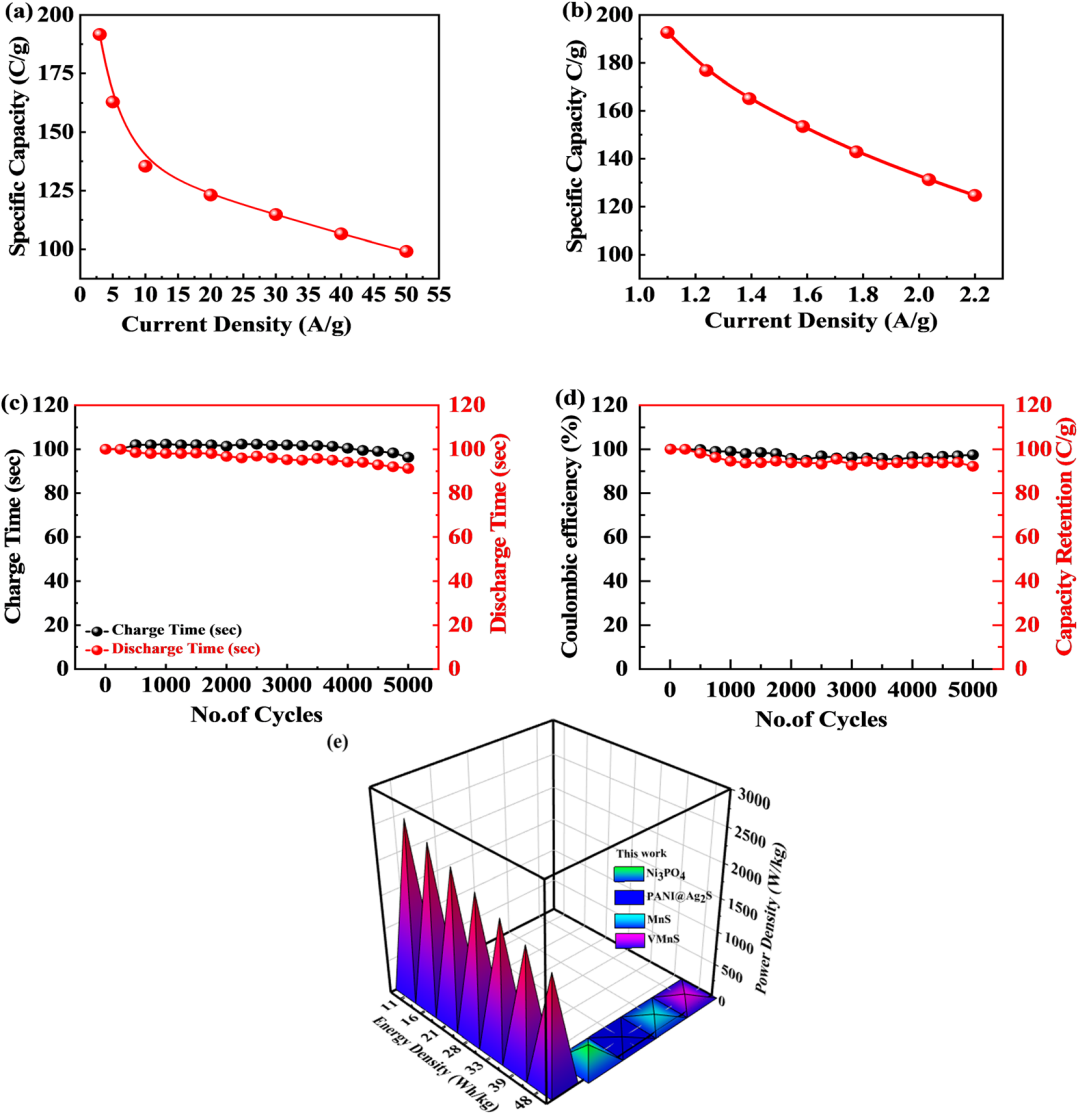
(a and b) Specific capacity for the S_2_//AC supercapattery computed through CV and GCD. (c and d) Charge/discharge and capacity retention for the S_2_//AC supercapattery after 5000 cycles. (e) Energy and power density for the S_2_//AC supercapattery.

The GCD measurements for the S_2_//AC supercapattery were performed at various densities (1.0 to 2.2 A g^−1^), as depicted in Fig. 10(d). The GCD curves of the S_2_//AC device were nearly symmetrical at all currents, which demonstrates its higher stability.^63^ Furthermore, the GCD contours were non-linear due to the faradaic response of S_2_. The specific capacity for the S_2_//AC supercapattery was also calculated from eqn (8) and (11). At a current intensity of 1.0 A g^−1^, the specific capacity of S_2_//AC was 193 C g^−1^, as indicated in Fig. 11(a). The S_2_//AC device can retain 60% of its initial capacity at 2.2 A g^−1^ current. While the capacity through a CV for the S_2_//AC supercapattery was 183 C g^−1^ (at 3 mV s^−1^), as indicated in Fig. 11(b).

Fig. 11(c) and (d) represent the stability test for the S_2_//AC supercapattery device against 5000 cycles. The capacity was increased marginally to 110% at the start, owing to the wettability of the electrode surface and slow activation of the electrode.^64^ After 5000 cycles, the capacity declined slowly to 92%, showing that the device was stable. The coulombic efficiency after 5000 cycles was retained at 98%. The decay in capacity was due to structural degradation of the electrode materials. The nanocomposite agglomeration as well as separation of the electrode material from the electrolyte are also responsible for the decrease in capacity.^65,66^

Fig. 11(e) depicts the Ragone plot of the estimated energy density (*E*) and power density (*P*) of the S_2_//AC supercapattery using the following formulae.14
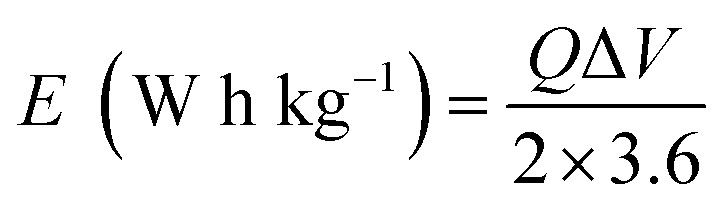
15
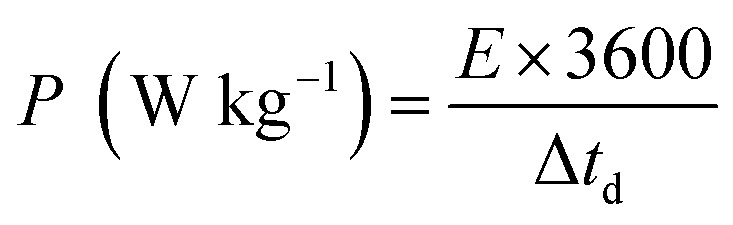
where *Q* denotes specific capacity, *V* denotes the PW, and *t*_d_ denotes discharge duration. S_2_//AC provided a high energy density as well as an outstanding power density (49.4 W h kg^−1^ at 550 W kg^−1^ and 19.5 W h kg^−1^ at 6382 W kg^−1^). These results outperformed previous studies, as indicated in Table 4.

**Table 4:** Energy *vs.* power density for the MgCuPO_4_–Ag_3_PO_4_//AC device and comparison with the literature

Previous study	Energy density	Power density
Ni–Co–S^70^	42	750
Ni–V–S_2_ (ref. 71)	12	900
MnS^72^	10.55	294.35
Sr-based materials^73^	21.8	224
Co/WS^74^	42.2	1047
2D VS_2_/P^75^	28.22	596
MnO_2_@CoS nanosheets^76^	34	597.24
**This work**	**49.4**	**550**

## 4. Oxygen evolution reaction (OER)

Linear sweep voltammetry (LSV) was employed to evaluate the electrocatalytic performance of MgCuPO_4_, S_1_, S_2_, S_3_, and S_4_ composites, as well as the bare stainless steel (SS) substrate. To conduct the investigation, 1 M KOH electrolyte was utilized. The primary objective was to assess their ability to generate a current density of 10 mA cm^−2^ with minimal overpotential, aiming to match the widely employed anode materials RuO_2_ and IrO_2_ in various industrial applications. The LSV data revealed distinctive behavior among the tested materials.^67^

16 *η* = *E*_RHE_ − 1.23 V

**Fig. 12:**
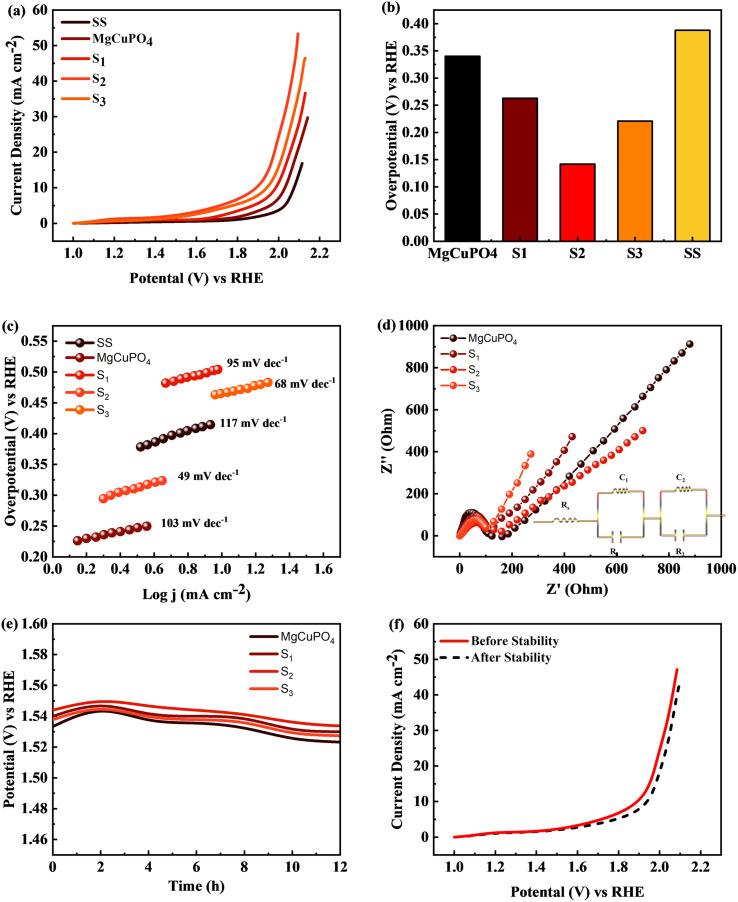
(a) Linear sweep voltammograms (LSV) of MgCuPO_4_, S_1_, S_2_, and S_3_ in comparison to SS. (b) Overpotential needed to achieve 10 mA cm^−2^ current for all composites. (c) Tafel plots for SS, MgCuPO_4_, S_1_, S_2_, and S_3_. (d) EIS measurements for MgCuPO_4_, S_1_, S_2_, and S_3_ composites. (e) Chronopotentiometric study for MgCuPO_4_, S_1_, S_2_, and S_3_. (f) Electrocatalytic analysis of S_2_ composite after OER stability.

The MgCuPO_4_ composite exhibited an onset overpotential of 340 mV, whereas the S_1_ and S_3_ composites displayed starting overpotentials of 263 and 221 mV, respectively. In contrast, the S_2_ composite showcased an even lower overpotential of 142 mV, further underscoring its electrocatalytic capabilities. Conversely, the bare SS substrate necessitated a higher overpotential of 388 mV to achieve a current density of 10 mA cm^−2^ (Fig. 12(a)).

The bare SS substrate was included in the assessment as a reference point to evaluate the effectiveness of the composites. The higher overpotential required by the bare SS substrate suggests low electrocatalytic activity and inadequate efficiency in the oxygen evolution reaction (OER). Conversely, the significantly lower overpotentials observed for the MgCuPO_4_, S_1_, S_2_, and S_3_ composites indicate superior performance and potential for diverse applications, particularly in renewable energy conversion and storage devices.

Moreover, Fig. 12(b) illustrates comparable overpotentials at *j* = 10 mA cm^−2^, a typical measure of OER activity. A lower overpotential implies significantly better performance in the OER.^68,69^ These findings highlight the exceptional electrocatalytic capabilities of the MgCuPO_4_, S_1_, S_2_ and S_3_ composites compared to the bare SS substrate. Their reduced onset overpotentials and improved catalyst utilization suggest the potential to enhance energy storage technologies and enable sustainable energy conversion processes. The effectiveness of the electrochemical processes can be improved by these materials (MgCuPO_4_, S_1_, S_2_, and S_3_), taking us one step forward to a more environmentally friendly and sustainable energy density.

Tafel plots were used to analyze the reaction kinetics of the MgCuPO_4_, S_1_, S_2_, and S_3_ materials, as shown in Fig. 12(c). The Tafel slopes reveal information about the reaction response of the materials.

17 *η* = *a* + *b* ln *i*

The MgCuPO_4_ composite exhibits a Tafel slope of 103 mV dec^−1^; the S_1_ composite shows a Tafel slope of 95 mV dec^−1^; S_2_ shows a Tafel slope of 49 mV dec^−1^ (see Table 5); and S_3_ shows a Tafel slope of 68 mV dec^−1^. The reduced Tafel slope of S_2_ (117 mV dec^−1^) in comparison to the other composites and bare SS points to quicker reaction rates. As a result, the oxygen evolution process (OER) can occur at greater speeds in the S_2_ composite due to its more effective catalytic activity.

**Table 5:** Comparison of Tafel slope and overpotential of this work with previous studies

Literature	Tafel slope (mV dec^−1^)	Overpotential (mV)	Ref.
Cobalt sulfide thin films	57	300	77
Ni_12_P_5_ nanocapsule	49.8	152	7
Ni_*x*_P_*y*_	72.2	157	78
C@Ni–P	64	153	79
**MgCuPO**_**4**_ **–Ag**_**3**_ **PO**_**4**_ **(S**_**2**_ **)**	**49**	**142**	**This work**

The charge transport process inside the MgCuPO_4_, S_1_, S_2_, and S_3_ materials, along with the bare SS substrate, was examined using electrochemical impedance spectra (EIS), in conjunction with Tafel studies. Significant features were discovered from the Nyquist plots, as illustrated in Fig. 12(d). Surprisingly, the semicircle diameter of the S_2_ composite was less than those of the MgCuPO_4_, S_1_, S_2_, and S_3_ composites. This suggests that the S_2_ composite is more conductible. As shown in the insets of Fig. 12(d), analogous circuits were used to analyze the EIS data. These circuits included elements that represented the impedance of the solution (*R*_s_), the inherent resistance (*R*_1_) of the catalyst, and the impedance of the electrocatalytic activity (*R*_2_). Notably, the electrocatalytic resistance of the S_2_ composite was determined to be 659 Ω cm^2^, significantly lower than the resistances of the MgCuPO_4_ (732 Ω cm^2^), S_1_ composite (788 Ω cm^2^) or the S_3_ composite (914 Ω cm^2^). Table S1† presents the impedance of MgCuPO_4_, S_1_, S_2_, and S_3_ in the oxygen evolution reaction.

To evaluate the long-term electrochemical stability of the MgCuPO_4_, S_1_, S_2_, and S_3_ composites, a chronopotentiometric mode was employed. Fig. 12(e) demonstrates a minor potential drop over time, attributed to an improvement in catalytic efficiency. Continuous gas liberation was observed during the stability investigation, indicating the ongoing oxygen evolution process. Fig. 12(f) presents the LSV graphs of S_2_.

The results of the study indicate that the MgCuPO_4_, S_1_, S_2_, and S_3_ composites exhibit favorable reaction kinetics and long-term electrochemical stability specifically for the oxygen evolution reaction (OER). The lower Tafel slope observed for the S_2_ composite suggests faster reaction rates, while the slight reduction in potential during the stability test signifies improved catalytic efficiency. These characteristics are crucial for ensuring the sustained performance and durability of electrochemical devices, underscoring the significance of these composites in applications related to energy storage and conversion.

## 5. Conclusions

The sonochemical and hydrothermal technique followed by calcination treatment effectively synthesizes MgCuPO_4_ and MgCuPO_4_–Ag_3_PO_4_ nanocomposites with various MgCuPO_4_–Ag_3_PO_4_ content ratios. XRD phase structure analysis verified that the crystalline structure of Ag_3_PO_4_ NPs increases with decreasing amorphous MgCuPO_4_ content. The FTIR spectra revealed that phosphate and hydroxyl groups were chemisorbed on the MgCuPO_4_–Ag_3_PO_4_ surface. The UV-vis spectroscopic analysis revealed that MgCuPO_4_–Ag_3_PO_4_ composites were more conductive than MgCuPO_4_. The SEM analysis showed that Ag_3_PO_4_ nanoparticles were firmly fixed on the amorphous surface of MgCuPO_4_. The MgCuPO_4_–Ag_3_PO_4_ nanocomposite-based electrode demonstrated increased rate capability from 53% (MgCuPO_4_) to 59% at 3.2 A g^−1^. The rapid electron movement and the large number of active sites given by MgCuPO_4_ and Ag_3_PO_4_ composites enhanced the rate capacity. Because amorphous MgCuPO_4_ has a low electrical conductivity, modifying amorphous MgCuPO_4_ with crystalline Ag_3_PO_4_ NPs offers a potential method to increase conductivity and rate capability. The constructed S_2_//AC supercapattery had an energy density of 49.4 W h kg^−1^ at 550 W kg^−1^ power density. The S_2_//AC supercapattery showed 92% capacity retention after 5000 cycles. Investigation of the OER application demonstrated that S_2_ exhibited the lowest Tafel slope, measuring 49 mV dec^−1^. The results obtained from this study suggest that MgCuPO_4_–Ag_3_PO_4_ composites hold great promise as potential candidates for future energy storage devices and further investigation into the oxygen evolution reaction (OER).

## Conflicts of interest

There are no conflicts to declare.

## Supplementary Material

NA-005-D3NA00466J-s001
