# Supplementary material for: Synergistic redox enhancement: silver phosphate augmentation for optimizing magnesium copper phosphate in efficient energy storage devices and oxygen evolution reaction
Source: Nanoscale Adv. 2023 Aug 7;5(18):4735–51. doi: 10.1039/d3na00466j (PMC10496879; doi:10.1039/d3na00466j)
Supplement: NA-005-D3NA00466J-s001 [file NA-005-D3NA00466J-s001.pdf]

Electronic Supplementary Material (ESI) for Nanoscale Advances.
This journal is © The Royal Society of Chemistry 2023

Synergistic Redox Enhancement: Silver Phosphate Augmentation for Optimizing Magnesium Copper Phosphate in Efficient Energy Storage Devices and Oxygen Evolution Reaction

Haseebul Hassan1, Muhammad Waqas Iqbal1*, Nora Hamad Al-Shaalan2, Sarah Alharthi3, Nawal D. Alqarni4, Mohammed A. Amin3, Amir Muhammad Afzal1

1Department of Physics, Riphah International University, Campus Lahore, Pakistan

2Department of Chemistry, College of Science, Princess Nourah bint Abdulrahman University, P.O. Box 84428, Riyadh 11671, Saudi Arabia

3Department of Chemistry, College of Science, Taif University, P.O. Box 11099, Taif

4Department of Chemistry, College of Science, University of Bisha, Bisha, 61922, Saudi Arabia

E-mail: waqas.iqbal@riphah.edu.pk,

Supplementary Section

In the supplementary part, we also operate the three-cell assembly in 0 – 0.8 working potential. As can be seen from Figure S1, as the operating potential increases the area under the CV curves decreases. The shape of the CV curves becomes more straight. This means that at the higher potential, the redox reactions did not occur. The charge stored is mainly due to the absorption/desorption process. This may be due to the insufficient time required for redox reactions. More the GCD curves did not show discharging. Thus we can conclude that the best operating potential for these materials ($MgCuPO_4$, S_1, S_2, and S_3) is 0 – 0.6 V. More the charge

storage in supercapattery was also checked through Dunn's model and the results were explained in Figure S_2.

$$i\,(v) = k_1 v + k_2 v^{0.5} \quad \textbf{(1)}$$

$$\frac{i(v)}{v^{0.5}} = k_1 v^{0.5} + k_2 \quad \textbf{(2)}$$

The above equations were used to determine the storage mechanism. K_1 indicates capacitive participation, and K_2 determines diffusive participation. As can be seen from Figure S_2, the capacitive contribution increases as the scan rate increases. At the first, the capacitive participation was 21% while diffusive participation was 79%. And as the scan rate increases to 50% the capacitive participation goes to 42%. This may be because, at the lower scan, the battery-graded electrode had enough time to complete the redox reactions. While at higher scan the battery-graded electrode had not enough time to complete the redox reactions. Table S1 represents the impedance of $MgCuPO_4$, S_1, S_2, and S_3 in oxygen evolution reaction.

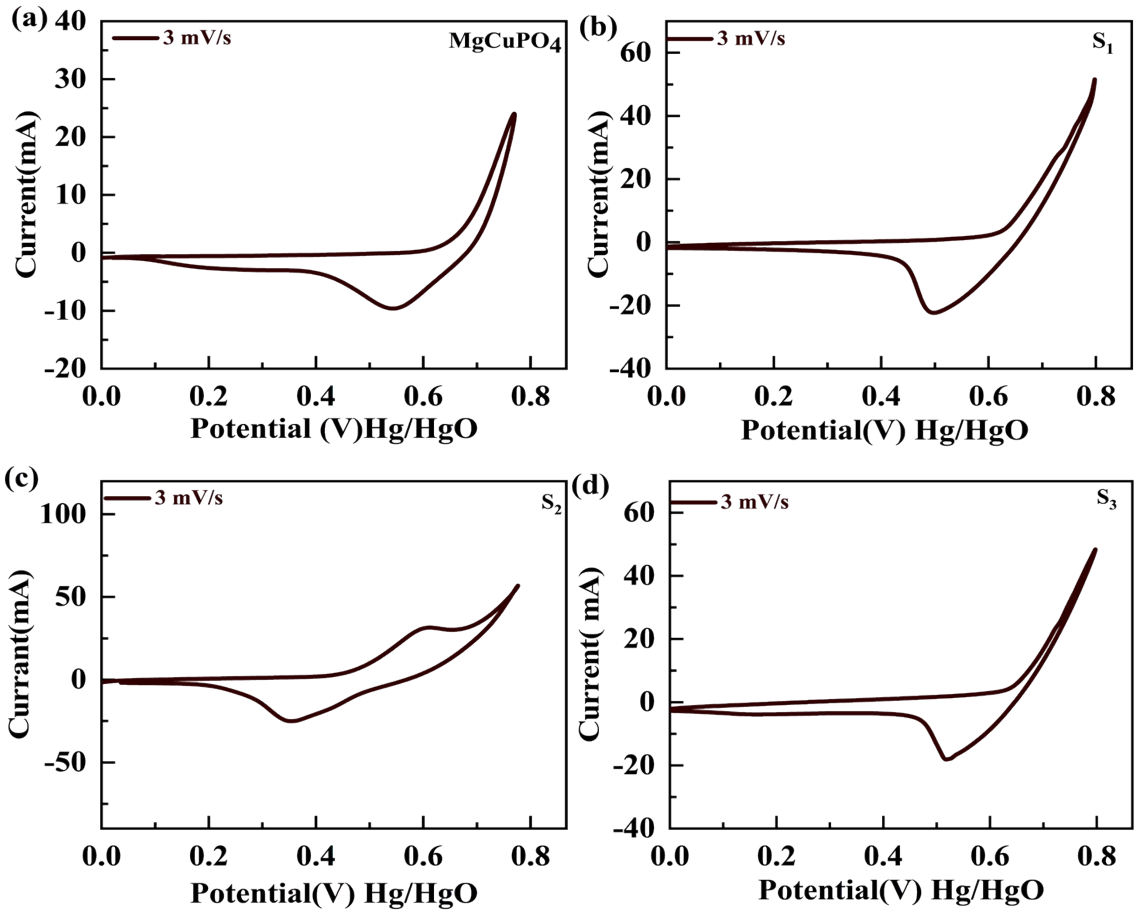

Figure S1. (a-d) Represents the CV curves for $MgCuPO_4$, S_1, S_2, and S_3 at operating potential from 0 – 0.8 V.

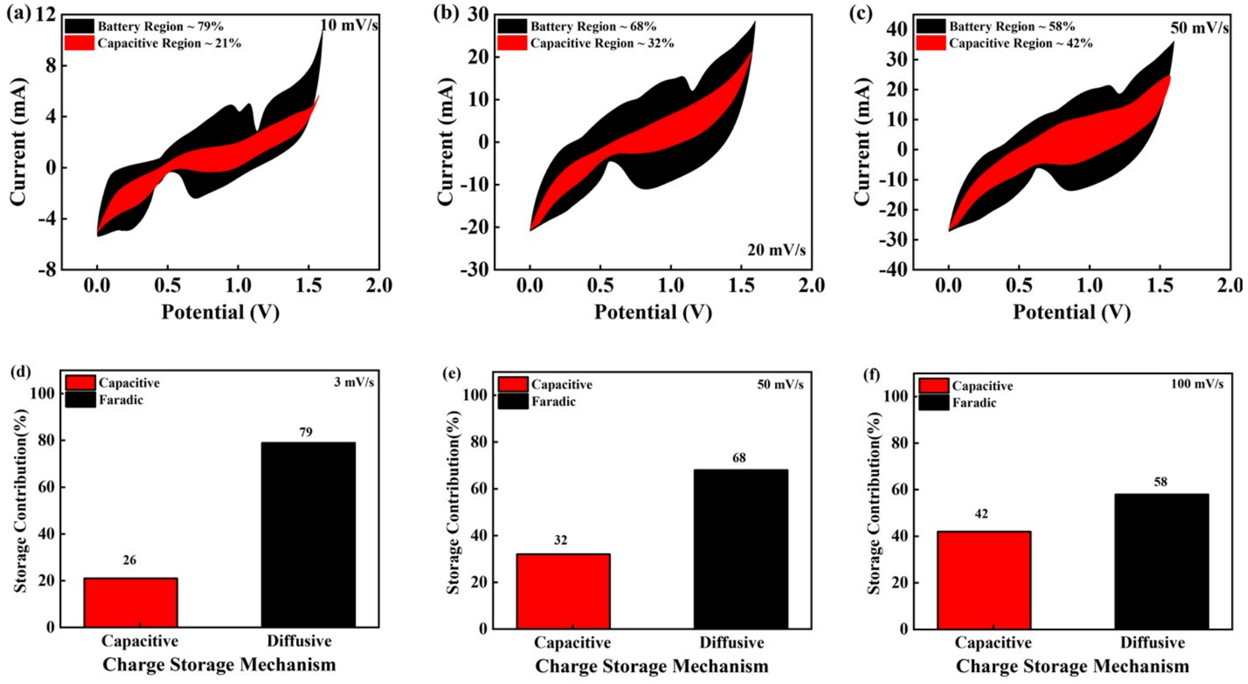

Figure S2. (a-c) Showed Dunn's model results at 10, 30, and 50 mV/s scan rate. (d-f) Bar chart showing the capacitive and diffusive participation.

**Materials**	**Impedance (Ω cm^2)**
$MgCuPO_4$	732
S_1	788
S_2	659
S_3	914

Table S1. Impedance measurements for $MgCuPO_4$, S_1, S_2, and S_3 in oxygen evolution reaction.
